# Unexpected Abscess Localization of the Anterior Abdominal Wall in an ADPKD Patient Undergoing Hemodialysis

**DOI:** 10.1155/2015/982575

**Published:** 2015-08-02

**Authors:** Nikos Sabanis, Eleni Paschou, Eleni Gavriilaki, Maria Mourounoglou, Sotirios Vasileiou

**Affiliations:** ^1^Department of Nephrology, General Hospital of Pella, 58200 Edessa, Greece; ^2^Department of General Practice and Family Medicine, General Hospital of Pella, 58200 Edessa, Greece; ^3^Medical School, Aristotle University of Thessaloniki, Thessaloniki, Greece; ^4^Department of General Surgery, General Hospital of Pella, 58200 Edessa, Greece

## Abstract

Autosomal Dominant Polycystic Kidney Disease (ADPKD) is one of the most common monogenic disorders and the leading inheritable cause of end-stage renal disease worldwide. Cystic and noncystic extrarenal manifestations are correlated with variable clinical presentations so that an inherited disorder is now considered a systemic disease. Kidney and liver cystic infections are the most common infectious complications in ADPKD patients. Furthermore, it is well known that ADPKD is commonly associated with colonic diverticular disease which recently has been reported to be linked to increased risk of infection on hemodialysis patients. Herein, we present a case of anterior abdominal wall abscess caused by *Enterococcus faecalis* in a patient with ADPKD undergoing hemodialysis. Although the precise pathway of infection remains uncertain, the previous medical history as well as the clinical course of our patient led us to hypothesize an alternative route of infection from the gastrointestinal tract through an aberrant intestinal barrier into the bloodstream and eventually to an atypical location.

## 1. Introduction

Autosomal Dominant Polycystic Kidney Disease (ADPKD) is the leading inheritable cause of end-stage renal disease affecting 1/500 to 1000 live births globally [1]. The progressive renal function impairment due to renal cyst formation is regarded as the cornerstone of the disease accounting for 5–10% of patients that require renal replacement therapy [2].

ADPKD is considered a systemic disease since not only are the kidneys affected but also multiorgan cystic and noncystic extrarenal features can occur [3]. Infectious complications in ADPKD patients, especially those undergoing hemodialysis, remain potential life-threatening consequences and tend to reoccur. Among extrarenal manifestations of polycystic kidney disease, colonic diverticulosis has been reported to be connected with infections in hemodialysis patients [4].

Herein, we present a case of an abscess located in the anterior abdominal wall, caused by* Enterococcus faecalis* in an ADPKD patient with colonic diverticular disease undergoing hemodialysis. We emphasize the importance of the predisposing factors in ADPKD patients that potentially promote bacterial migration from intestinal tract via the bloodstream to other tissues and organs.

To the best of our knowledge, this is the first report in the literature of an abscess of abdominal wall in an ADPKD patient receiving hemodialysis.

## 2. Case Presentation

A 67-year-old Caucasian male patient was admitted to emergency department due to abdominal pain lasting two days, accompanied with weakness, chills, and high-grade fever. He was a nonsmoker, retired state employee and drank alcohol only occasionally. He had not travelled abroad and no contact with domestic animals was mentioned on routine questioning. On physical examination, the patient was pale and confused and his temperature was 40.1°C. We observed tachypnea with respiratory rate 30/min and SpO_2_ 98% while lung auscultation was normal. Heart rate was 110 beats per minute and his blood pressure measured 100/60 mmHg. The remaining physical examination revealed signs of skin inflammation of the upper left abdominal quadrant including redness, smooth swelling, warmth, and tenderness. No inflammation signs of the arteriovenous fistula or skin wounds were observed. The liver was palpable 6 cm below the right costal margin. Digital rectal examination was negative. No other signs compatible with diverticulosis were recognized.

Initial laboratory examinations revealed leukocytosis (white blood cells 12.7 × 10^3^/*μ*L) and neutrophilia (87.3%), anemia (hematocrit 30.6%, hemoglobin 10 g/dL), and highly increased inflammation markers (C-reactive protein 55.1 mg/dL with normal range 0–0.5 mg/dL, and Erythrocyte Sedimentation Rate 98 mm/h).

The patient had a medical history of hypertension and ADPKD undergoing hemodialysis through arteriovenous fistula during the last 15 years and his father was also an ADPKD patient. In the past, the patient had experienced recurrent episodes of gross hematuria and nephrolithiasis complicated with urinary tract infections (UTI). Two months before, the patient had been hospitalized due to* Escherichia coli* UTI, without further imaging findings of cyst infection, receiving ciprofloxacin treatment based on antibiogram susceptibility. During the last month, he also encountered severe episodes of intradialytic hypotension that required reassessment of dry weight and discontinuation of antihypertensive therapy.

**Figure 1:**
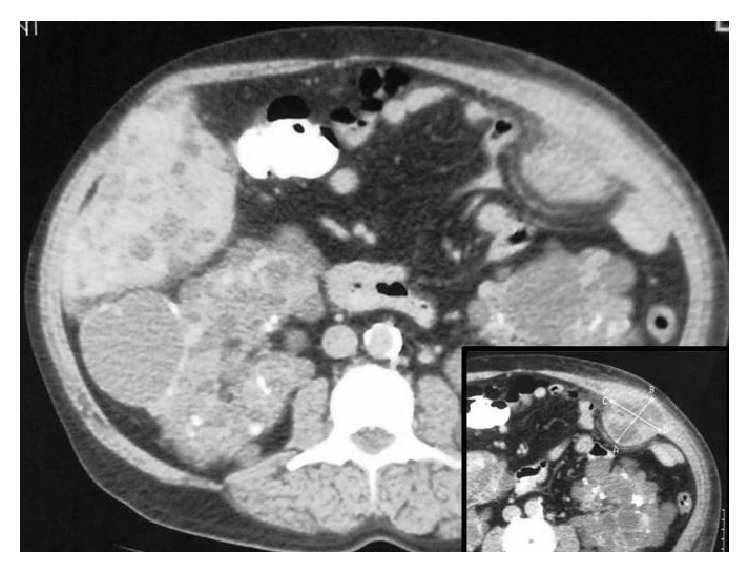
Abdominal Computed Tomography: abscess of the left anterior abdominal wall.

**Figure 2:**
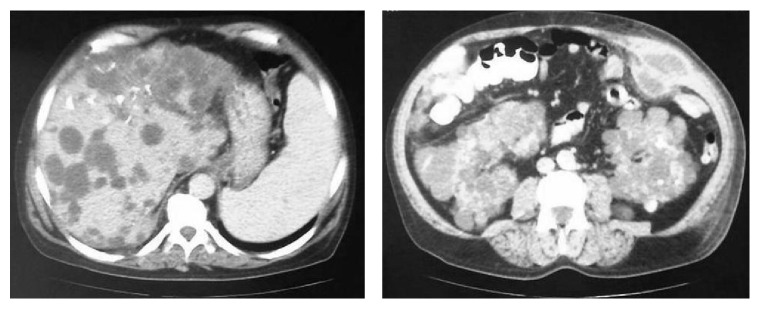
Abdominal Computed Tomography: diffuse involvement of polycystic disease in both kidneys, liver, and colon.

Abdominal Computed Tomography showed an extensive, well-limited abscess of the left anterior abdominal wall with dimensions 5.7 × 5.5 cm (Figure 1), splenomegaly, multiple kidney and liver cysts, and colonic diverticulosis with no evidence of active infection (Figure 2).

The patient underwent immediate surgical drainage and the cultures revealed non-vancomycin resistant* Enterococcus faecalis*. Blood and urine cultures were negative. Transthoracic echocardiography revealed no evidence of infective endocarditis. He received antibiotic treatment with linezolid in a dose of 600 mg twice daily and metronidazole 500 mg twice daily for 12 days in total. After surgical intervention, the patient's clinical course remained uneventful and he was discharged home on the 13th day.

## 3. Discussion

Autosomal Dominant Polycystic Kidney Disease is considered a systemic disease with diverse cystic and noncystic manifestations that can lead to increased morbidity and mortality [5]. Infectious complications are frequently related to cyst infections and remain potentially a life-threatening outcome [6]. In general, the route of renal and liver cyst infections in ADPKD patients remains questionable [7, 8] although the retrograde pathway from the bladder or the biliary tree prospectively has been proposed as a driving-force mechanism.

Moreover, ADPKD patients with increased intra-abdominal pressure due to kidneys and liver cystic enlargement are characterized by increased risk of cyst infections [9] possibly due to an additional reduction of venous return leading to impaired cardiac output and as a result of intestinal ischemia. Thus, intra-abdominal hypertension can significantly increase the intestinal mucosa permeability through reduction of microcirculation blood flow leading to bacterial or endotoxin translocation [10, 11]. Finally, essential alterations in the function and structure of intestinal smooth muscle cells as a direct effect of PKD1/PKD2 mutations may increase the incidence of bacterial translocation [12–14].

The key role of bacterial translocation as an alternative pathway related to infections has not been elucidated even though the colon diverticular disease has been correlated with increased risk [4]. In addition, recent studies have focused on the close relationship between the kidney and the gastrointestinal track, ordinarily referred to as “kidney-gut axis” [15]. Hence, bacterial translocation depicts the epiphenomenon of a complicated interplay between the human gut microflora via aberrant epithelial barrier and the uremic milieu [16].

The presence of end-stage renal disease (ESRD) has been correlated with structural and functional alterations of the intestinal mucosa barrier and important changes of gut microbiota composition and development [17]. As a result, intact bacteria or bacterial bioproducts migrate from the intestinal lumen into systemic circulation. In this regard, the appearance of unusual infections in remote locations could be related to bacterial translocation. In hemodialysis patients, the high risk of bacterial translocation has been associated with various predisposing factors such as the prolonged colonic transit time due to dietary restrictions and the epithelial wall edema due to heart failure [18]. The use of phosphate binders and wide range antibiotics deteriorates the reduced intestinal motility and worsens the vulnerable balance of the intestinal ecosystem, respectively [19]. In the same context, jeopardized perfusion during recurrent episodes of intradialytic hypotension alters the transepithelial resistance.

Our patient was an ADPKD patient with increased abdominal pressure due to enlarged kidney and liver cysts. The coexistence of diverticular colon disease, persistent constipation, and the previous ciprofloxacin treatment that resulted in derangement of gut microflora in combination with recurrent episodes of intradialytic hypotension led us to assume that bacterial translocation could explain the uncommon abscess localization. The fact that no other inflammation outbreaks were recognized and especially the absence of diverticulitis could support our hypothesis.

## 4. Conclusion

Infectious complications in ADPKD patients are usually referred to hepatic or renal cystic infections and the retrograde path has been considered the leading pathophysiological mechanism. The potential role of bacterial translocation as an alternative pathway has only been described in ADPKD patient with diverticular disease. In our case, we focus on the coexistence of diverse predisposing factors that enhance bacterial translocation and may explain the unexpected localization of an abscess in the abdominal wall caused by a pathogen of intestinal microbiota.
